# Children’s cognitive impairment associated with cassava cyanide in Democratic Republic of the Congo: Burden of disease

**DOI:** 10.1371/journal.pgph.0002761

**Published:** 2024-01-16

**Authors:** Chen Chen, Felicia Wu

**Affiliations:** 1 School of Public Health, Shandong University, Jinan, Shandong Province, China; 2 Department of Food Science & Human Nutrition, Michigan State University, East Lansing, Michigan, United States of America; 3 Agricultural, Food, and Resource Economics, Michigan State University, East Lansing, Michigan, United States of America; University of Ghana School of Public Health, GHANA

## Abstract

Worldwide, tens of millions of children rely on cassava as a dietary staple throughout their formative years of brain and behavioral development. Recently, it was discovered that cassava cyanide can impair children’s neurocognitive development at relatively low exposures. We revisited the World Health Organization’s foodborne disease burden estimate of cassava cyanide, focusing on the new health endpoint of children’s cognitive impairment in Democratic Republic of the Congo (DRC). The loss of Intelligence Quotient (IQ) scores was used to measure the endpoint of cognitive impairment caused by cassava cyanide exposure, which was estimated based on the concentration-effect relationship between children’s IQ scores and cyanide concentrations in flour. We estimated the burden of intellectual disability (ID) associated with cassava cyanide exposure in terms of disability-adjusted life years (DALYs). The median content of cyanide in cassava samples collected from DRC was 12.5 mg/kg, causing a median decrement to children’s IQ of 2.37 points. The estimated number of children with ID associated with cassava cyanide exposure was 1,643 cases, although 1,567 of these cases (95%) were mild ID. The burden of cognitive impairment attributable to cassava cyanide in DRC alone was 13,862 DALYs per 100,000 children, or 3.01 million for all children under age 5. The results of the study, showing a significant burden of cassava cyanide-related cognitive impairment in children even at relatively low doses, can contribute to the implementation of cost-effective interventions to make cassava consumption safer for children in high-risk rural areas of DRC.

## Introduction

Bitter cassava, a dietary staple for over 600 million people worldwide, is grown and consumed throughout Central Africa, South America, East Asia, and Pacific Island countries. Cassava contains cyanogenic glycosides, primarily linamarin, that can be deadly at high doses and cause chronic diseases in humans [1]. Konzo, a non-progressive but sudden-onset paralytic disease primarily affecting the legs, has been associated with cyanide exposure from a monotonous diet of bitter cassava in Africa, with many cases reported in Democratic Republic of the Congo [2]. Recent studies indicate that there is an association between konzo and reduced cognitive function, particularly in children. In DR Congo, cognitive impairment was detected in children in konzo-endemic regions, even if they do not have konzo; implying that in high-risk regions, exposures to cassava cyanide that may not cause konzo can still impair children’s neurodevelopment. Otherwise healthy schoolchildren living in konzo-endemic areas present with cognitive impairment compared to children from non-konzo-endemic areas [3]. A long-term decline in cognitive abilities has been observed in children who were exposed to cyanide, based on a prospective cohort study [4]. The latest research has expanded on these findings, indicating that the harmful effects of cassava consumption may commence when children transition from breastfeeding to consuming cassava porridge, even as early as one year old [5]. Outbreaks of konzo are common in Central African regions with drought and famine. Recently, Zambia, a country previously had not been affected by konzo, reported its first cases [6,7].

Bitter cassava is typically processed to reduce cyanide before consumption. Pounding, soaking, sun drying, and fermentation are traditional methods to accomplish this [1]. In Central Africa, cassava roots are soaked and pounded to produce flour used for porridge. However, these processing methods are not always sufficient to prevent cyanide intoxication, especially during drought conditions that may both increase cyanide concentrations in cassava and exacerbate hunger [8]. The wetting method is a food processing technique to further reduce cyanide: mixing cassava flour with water, then spreading the mixture in a thin layer to break down the cyanogens [9]. In humans, the detoxification process of cyanide by conversion to thiocyanates requires sulfur-containing amino acids. Insufficient protein intake in the diet increases cyanide levels in the blood, which impairs cyanide detoxification and likely triggers konzo [10,11].

Prolonged intake of insufficiently processed bitter cassava roots or cassava juice containing linamarin induced neurotoxic effects and locomotor impairment in rats [12–14]. As now there is suggestive evidence that cassava cyanide is associated with cognitive impairment among children with konzo, although a concentration-effect relationship has not been established yet. It is important to reassess the disease burden caused by cassava cyanide exposure. The World Health Organization (WHO) published a comprehensive report on the global burden of foodborne disease, which included cassava cyanide as one of multiple food contaminants examined, focusing on six nations in Africa [15]. The only adverse health effect assessed for cassava was konzo. Konzo can result in permanent disability, with a correspondingly high disease burden of 1,066 cases per year, 227 deaths, and a total of 18,203 disability-adjusted life years (DALYs) per year [16]. However, cassava cyanide ranked relatively low among food contaminants for contributing to global disease burden [17]. Children whose cassava cyanide exposure may not trigger konzo may still suffer cognitive impairment persisting for years [2,4], which could dramatically increase the true disease burden. Thus, the neurocognitive effects documented for non-konzo children in konzo-affected communities make it more important to ensure food safety in regions dependent on bitter cassava with high levels of cyanogenic compounds. Hence, a new estimate for the disease burden is required for this food contaminant that accounts for not just the health endpoint of konzo, but also of neurological deficits.

## Material and methods

### Literature search for the occurrence and effects of cyanide in cassava

To determine health effects beyond konzo that have been associated with cassava cyanide exposure, as well as human exposures globally, we conducted a systematic literature search in PubMed and Google Scholar database using the keywords of (cassava Congo) OR (cyanogen Congo) OR (cyanide Congo) OR (cyanogenic glycosides Congo). Additionally, the monograph of the 74^th^ Joint FAO/WHO Expert Committee on Food Additives (JECFA) meeting and relevant references on cyanogenic glycosides was consulted [1].

**Fig 1:**
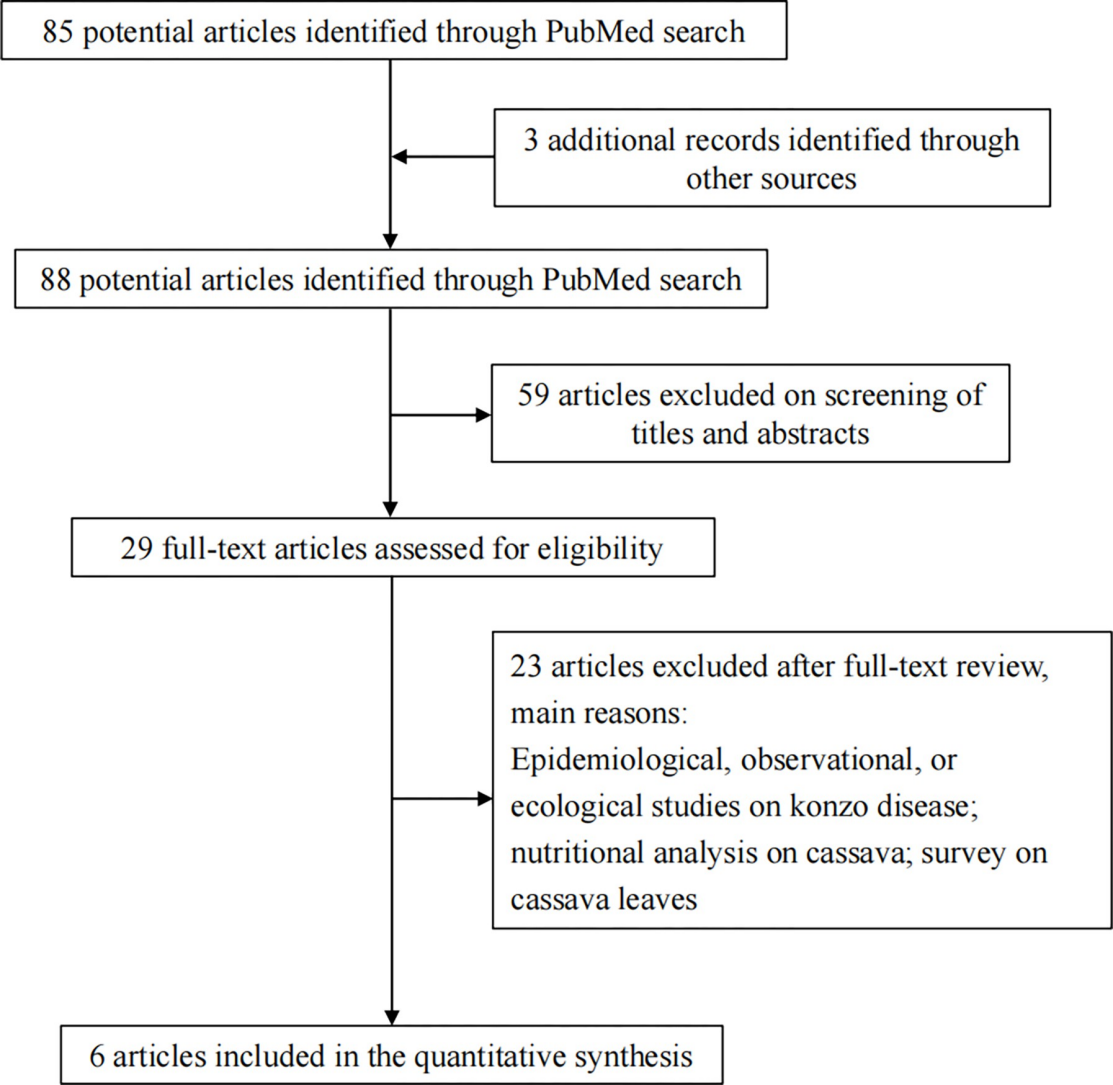
Flowchart for the literature search on cassava cyanide and children’s cognitive impairment.

The flowchart of the literature search process is shown in **Fig 1**. We retrieved 85 studies based on the search terms, and 3 additional articles were identified from other sources. After applying the eligibility criteria described above at the title and abstract level, 59 studies were excluded. We read the full texts of these studies, from which 6 were selected with sufficient data for our analysis.

### Critical endpoints of disease

Disease burden estimates associated with food contaminants require a clear definition of the health endpoint. Cognitive impairment has recently been found to be associated with poorly processed cassava in a children’s cohort in DRC, with quantitative measures of association elucidated [4]. Therefore, we chose the health endpoint of children’s cognitive impairment associated with cyanide exposure [5]. The Mullen Scales of Early Learning (MSEL) is a standardized developmental assessment that measures delays and disabilities in motor and cognitive development for children under age 5 in psychological settings [18,19]. The Cognitive Ability Composite (CAC) score provides the general score characterizing an overall cognitive performance of children, which is equivalent to the traditional IQ score. We used the Mullen CAC score as a measure for the endpoint. We did not include tropical ataxic neuropathy, as the evidence linking cassava cyanide exposure with this condition is less quantitatively established. Nor do we include konzo, which was already assessed by WHO.

### Incidence of intellectual disability

The MSEL CAC standardized score is equivalent to the traditional IQ (intelligence quotient) score [20]. To estimate disease burden from cassava cyanide exposure in DRC, intellectual disability (ID) was the selected health endpoint, as WHO has provided guidelines and disability weights for evaluating this specific endpoint. We assume that CAC scores have an average of 100 in the absence of cyanide exposure and a standard deviation of 15. CAC scores below 70 were considered as intellectually disabled. We calculated the expected changes in the number of children below CAC (IQ) score of 70 caused by the shift in the normal distribution, as a result of dietary cyanide exposure. Then the cases that within each of the ID categories were calculated on the basis of the classification of the CAC scores: mild (50–70), moderate (35–50), severe (20–35), and profound (<20) [21].

### Concentration-effect relationship between cyanide exposure and children’s CAC scores

Several epidemiological studies have assessed the relationship between cyanide exposure and cognitive development in Congolese children [2,4,5]. For this analysis, the concentration-effect relationship between cyanide exposure and children’s CAC scores was generated from one study in which concentrations of cyanogens in household cassava flour were significantly associated with cognitive impairment in Congolese children [5]. Mullen scales were used to assess cognitive development, and T-scores from these scales were combined into an Early Learning Composite (ELC) score, characterizing overall cognitive performance. On the basis of multivariable analyses, child neurodevelopment was found to be the main variable significantly associated with cyanide concentrations in cassava flour, with a coefficient of -0.19 (p < 0.01) after adjusting for biological and socioeconomic variables in households without konzo [5]. Hence, in this analysis, we used the estimate that every additional 1 mg/kg of cyanogen in cassava flour results in a 0.19 loss in CAC score.

### Estimating burden of cassava cyanide-related disease in DRC

The burden caused by a disease is measured in terms of both premature mortality caused by that disease, and years of morbidity. These concepts are neatly captured in the health-economic metric DALYs. DALYs due to ID associated with cassava cyanide exposure were calculated according to the WHO methodology [22]. The DALY extends the concept of years of life lost due to premature death to include equivalent years of “healthy” life lost in states of suboptimal health, broadly termed disability [23]. One DALY can be considered one lost year of optimally healthy life. The total number of DALYs caused by a disease is the sum of the years of life lost due to mortality (YLL) from the disease, and the years lived with disability (YLD) from that disease multiplied by a disability weight (dw) between 0 and 1, depending on the severity of the disability. Summed across a population:

DALY=YLL+YLD*dw [1]

In this case, the fatality rate of intellectual disability is assumed to be zero; therefore, only YLDs contribute to the total DALYs. The YLDs are estimated by the product of the category-specific number of incident cases, duration, and disability weight. Total ID YLDs are obtained by summing the category-specific YLDs. We assume the age-of-onset of intellectual impairment caused by cyanide exposure starts from weaning age (about 24 months), and the resulting outcome is assumed to be lifelong. Therefore, the duration of intellectual impairment lasts for the life expectancy at the age of two year. The life expectancy at the age of two (64.57 years old), and the population size of children under age 5 for 2020 (21.7 million) in DRC was derived from World Population Prospects [24]. We used the distribution of cyanide concentrations in cassava flour to calculate the percentages of children with ID in DRC. The disability weights for each ID category were adopted from the WHO Global Health Estimates study: 0.127 for mild ID, 0.293 for moderate ID, 0.383 for severe ID, and 0.444 for profound ID [22]. On the basis of these disability weights, we applied the composite disability weight to the DALY equation to estimate the burden of ID associated with cassava cyanide.

### Estimation of disease burden through Monte Carlo simulation

On the basis of the literature search, the distributions of cyanogen contents in cassava flour in DRC are generally skewed, so the estimates were characterized with lognormal distributions with geometric mean (GM) and geometric standard deviation (GSD). The GM values were calculated from mean cyanogen contents from various studies (Table 1). The estimate for GSD was calculated as the following equation:

$$GSD=exp\;[sqrt\;(ln\;(CV^{2}+1))]$$ [2]

where CV stands for the coefficient of variation and it was calculated by dividing the standard deviation by the mean value of the cyanide distribution.

**Table 1:** Summary of studies showing hydrogen cyanide (HCN) concentrations in cassava flour or processed cassava in the Democratic Republic of the Congo (DRC).

Cassava products	Location of sampling	Year	Number/type of samples	Mean HCN equivalent concentration (range, standard deviation) (mg/kg)*	Reference
Cassava cossettes	Five markets (Ngaba, Lemba, Livulu, RondPoint, and Matete) of Kinshasa	Not specified	Cossettes; 5 different areas, 3 samples each	2.8±0.4; 1.7±0.2; 1.5±0.2; 1.6±0.4; 2.9±0.5	Ngudi et al. 2002 [28]
Cassava flour	Six villages in Boko (Makiku, Kitati, Kipesi, Mangungu, Mutombo, Kinkamba)	2014	30 samples from each village	Before using wetting method: 19, 20, 20, 22, 41, 41; after using wetting method: 8, 8, 9, 9, 11, 12	Banea et al. 2015 [26]
Cassava roots, processed	Burhinyi of the Eastern Province of South-Kivu	2003, 2005	21 samples from the patients’ household	20 (5–300, SD = 73)	Chabwine et al. 2011 [27]
Cassava flour	Three Boko villages (Imboso Mwanga, Ikusama and Ikialala)	July 2011 (baseline), Nov 2011, Feb 2012, Jul 2012 (during intervention)	30 samples from each village	Four time intervals: 44 (SD = 38), 74 (75), 43 (50); 18 (19), 31 (26), 38 (38); 21 (19), 20 (16), 14 (16); 15(15), 18(16), 9(11)	Banea et al. 2013 [32]
Cassava flour	Kay Kalenge village	Mar 2010 to Sep 2011; revisited in Nov 2012	30 samples	After intervention: 9 (SD = 4)	Banea et al. 2014 [31]
Cassava flour	Four villages (Kay Kalenge, Indaba, Bilungu and Munkoki)	Before using wetting: Aug 2010; after using wetting method: Dec 2010, May 2011, Sep 2011	9 samples before and 15−30 samples after wetting method	Before: 22 (SD = 12); after: 4(8), 8(7), 7(5)	Banea et al. 2012 [30]

^*^SD denotes standard deviation.

To calculate the statistical estimates of the children’s CAC attributable to cyanide exposure from cassava in DRC, the exposure distribution models were run with a two-dimensional Monte Carlo simulation, representing variability and uncertainty in separate dimensions. For each country where the occurrence of cyanide in cassava flour is available, 10,000 iterations were performed for variability and 1,000 iterations for uncertainty. Simulations were performed by the statistical software R.

## Results

### Occurrence of cyanide in cassava flour

Studies showing the content of cyanogenic glycosides in cassava flour in DRC are listed in **Table 1**. The total levels of cyanogens in fresh bitter cassava roots typically are in the range of 100–500 mg hydrogen cyanide (HCN) equivalents (eq)/kg in DRC, and even above 1000 mg HCN eq/kg [25]. Processing cassava roots can significantly reduce the cyanogen contents; the mean cyanogen levels ranged from 1.45 to 20 mg HCN eq/kg in cassava food such as cossettes and gari [26–29]. Recently, a wetting method has been proved to be an effective intervention in reducing levels of cyanogens in cassava flour in DRC. For example, significant reductions of cyanide content were observed in different villages in Boko of DRC: the mean cyanide content in cassava flour reduced from 19–41 to 8–12 mg HCN eq/kg during an intervention program, and most of the samples were below the WHO safe limit of 10 mg/kg [26,30,31]. The estimated median content of cyanide in cassava flour was 12.5 mg/kg.

### CAC decrements (ΔCAC) attributable to cassava cyanide exposure

**Table 2** shows the average expected CAC decrements (ΔCAC) attributable to cassava cyanide exposure in children, with percentiles based on different levels of cyanide contents in cassava flour. The estimated average CAC decrement was 3.12 points in children population. The median estimate of CAC decrement was 2.37 points at the median and increased to 8.02 points at 95th percentile.

**Table 2:** Average and percentiles (90% confidence interval) of cyanide contents in cassava flour, the estimated CAC decrements (ΔCAC), and the estimated loss of DALYs attributable to dietary cyanide exposure from cassava flour.

**Average and percentiles of cyanide contents in cassava flour in DRC**					
Average	10th percentile	Median	90th percentile	95th percentile	99th percentile
16.42 (0,16.5)	4.84 (0,4.87)	12.49 (0,12.55)	32.24 (0,32.47)	42.19 (0,42.55)	69.8 (0,70.88)
**Estimated CAC decrements (ΔCAC) attributable to dietary cyanide exposure**					
Average	10th percentile	Median	90th percentile	95th percentile	99th percentile
3.12 (0,3.13)	0.92 (0,0.93)	2.37 (0,2.38)	6.13 (0,6.17)	8.02 (0,8.08)	13.26 (0,13.47)
**Estimated median disease burden in DALYs**					
Mild ID	Moderate ID	Severe ID	Profound ID	Total	
12,452 (0,12,937)	1,338 (0,1,613)	72 (0,168)	0 (0,56)	13,862 (0,14,773)	

### Estimated cases of children with intellectual disability

**Fig 2:**
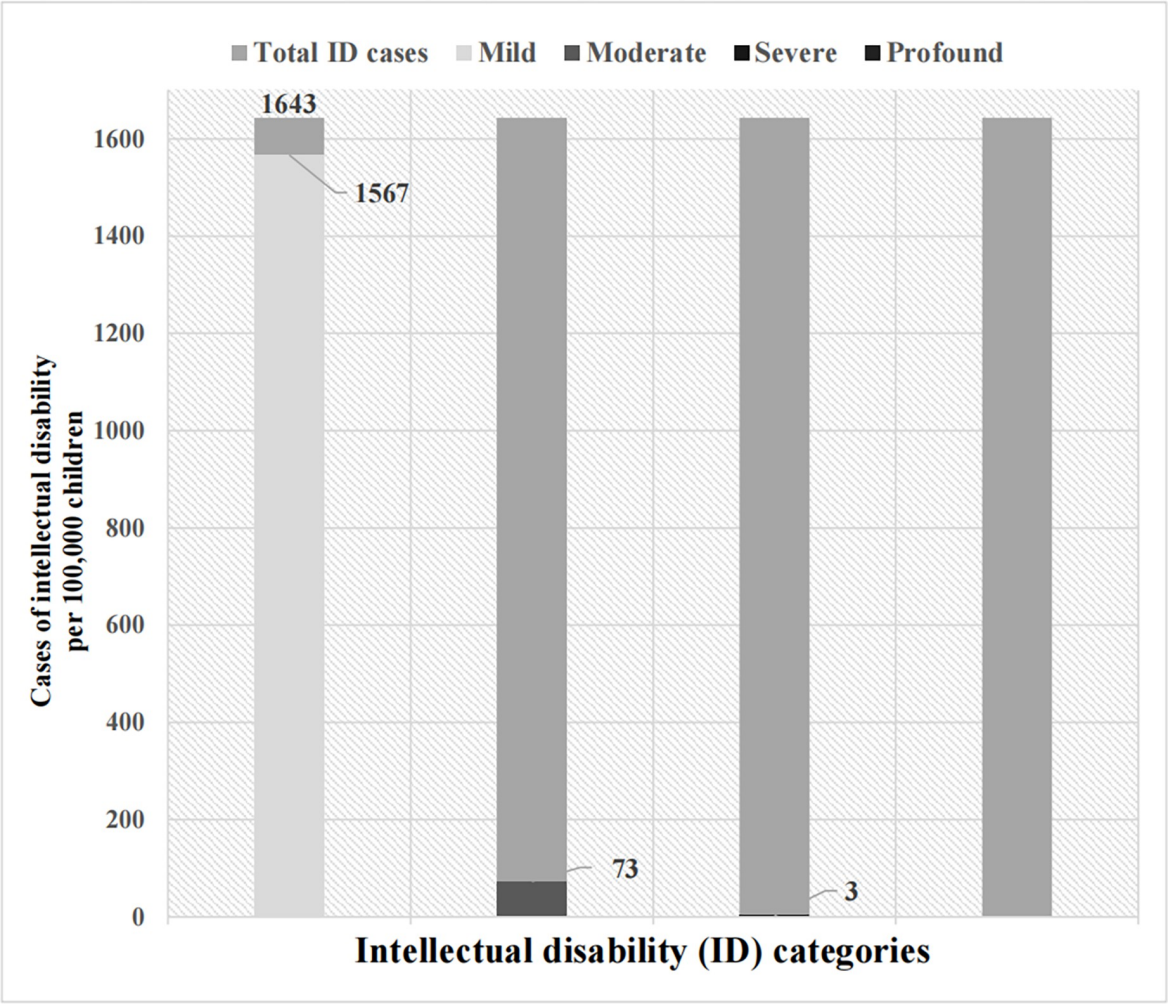
The change of individual cases with intellectual disability attributable to dietary exposure of cassava cyanide every 100,000 children.

The change of cases of individual children with mild, moderate, severe, or profound ID attributable to dietary exposure of cyanide were calculated. The median incidence of mild (50<CAC<70), moderate (35<CAC<50), and severe ID (20<CAC<35) cognitive impairment was 1,567 cases, 73 cases, and 3 cases respectively in DRC every 100,000 children (**Fig 2**). There was no incidence of profound ID (CAC<20) caused by cyanide exposure among Congolese children.

### Burden of cassava cyanide-related disease in DRC

On the basis of the potential years of life lost due to intellectual disability and life expectancy, we estimated the DALYs attributable to dietary exposure of cassava cyanide, which were calculated as the cases in each ID category and the total cases using age 2 as the time of onset for intellectual impairment. In DRC, the decrement of 2.37 CAC points resulted in the total number of DALYs at 13,862 (95% CI: 0, 14,773) every 100,000 children or 3.01 million for all children under age 5 in DRC (Table 2).

A report associated with the WHO Global Burden of Foodborne Disease calculated the total number of konzo cases and burden of disease associated with konzo in the six nations in Africa (Angola, Cameroon, Central African Republic, DRC, Mozambique, and Tanzania), which is 18,203 DALYs per year, and DRC contributed most to the disease burden [16]. However, these DALY estimates were calculated based on annually reported incidence cases caused by the foodborne chemicals, which may underestimate the true incidence of the disease and are different from the present study that applied a concentration-effect relationship between cyanide exposure and the health outcome of interest. The aflatoxin estimate was based not on incidence, but on quantitative cancer risk assessment and population attributable risk [33,34].

## Discussion

Initially, konzo was characterized by the WHO as an upper-motor neuron disease confined to motor pathways in the central nervous system (CNS). Because it has been known for decades that cassava cyanide causes konzo, it was assumed that cassava cyanide’s main effects were to the CNS, and that cognitive effects were minimal. In addition, cassava cyanide was associated with tropical ataxic neuropathy (TAN); although some studies cast doubt on that association, or link it with malnutritional risk factors such as deficiencies of thiamine, sulfur amino acids, and B vitamins [35]. Neuropsychological findings suggested that children in konzo-affected communities fail to improve the neurocognitive performance and motor proficiency later in their lives [4]. Recent findings have also shown that even children as young as one year old may experience neurodevelopmental issues when transitioning from breast milk to a diet primarily based on cassava porridge [5]. In addition, subclinical symptoms have been observed in children living in konzo-affected households, suggesting the existence of a pre-konzo condition that serves as a warning sign before the disease fully manifests [3]. Evidence on neuropathology has demonstrated a significant impairment in the pyramidal system function. Additionally, there is evidence of subclinical involvement of sensory pathways in the affected individuals. Furthermore, non-epileptic electroencephalographic abnormalities have been detected, while magnetic resonance imaging of the subjects have not shown any notable abnormalities [36].

Our estimates of the burden of cassava cyanide-associated cognitive impairment are similar to that of caused by dietary lead which is estimated at 5.2 million at the global scale. Carrington et al. (2019) investigated the global burden of disease due to dietary lead exposure on the basis of the exposure distributions and dose-response models [21]. In the Western Pacific Region B, children’s CAC decrements and the total number of ID cases attributable to dietary lead exposure were estimated to be 2.12 points and 1,117 cases: comparable to the burden of cognitive impairment associated with cassava cyanide (with 2.37 points of CAC decrements and 1,643 cases of ID) in DRC. By comparison, the global burden of ID cases resulting from prenatal exposure to methylmercury was lower, with estimated DALY of 1.96 million [37].

New evidence is emerging that adverse effects other than konzo are caused by dietary cassava cyanide exposure. Electrophysiological evidence has emerged suggesting that higher-level brain functioning may be affected as well [38]. Then, in recent longitudinal studies in the DRC using specialized neurocognitive tests of memory and learning in school-age children, pervasive and significant neurocognitive effects of cassava cyanide exposure in children who had mild or undetectable neurological symptoms were found. These subtler symptoms may constitute a pre-konzo condition, providing a warning that a child is approaching the disease’s threshold for neurocognitive effects [36]. A recent WHO report estimated global burden of foodborne diseases, including cassava cyanide; but only included konzo as the health outcome [15]. In the present study, we extended the estimates of the burden of disease associated with cognitive impairment among Congolese children. Our neuropsychological assessment can add to the current classification scheme of konzo (including disability, functioning, and health) by bridging the burden of the disease with the neurocognitive development of children.

This study has limitations. Firstly, based on the literature search, relevant studies were mainly conducted in province of Bandundu and South-Kivu where konzo epidemics have been reported, whereas surveys from other areas are limited. As a result, the burden of intellectual disability attributable to cyanide exposure from cassava consumption varies substantially among different locations within the country, because of differences in cyanide exposure due to processing methods, diet quality, and cassava varieties. More comprehensive surveys are recommended in other potentially high-risk locations in the future. Secondly, a dose-response relationship between cyanide exposure and the neurological effect has not yet been established. In this work, we generated the concentration-effect relationship between cyanide content in cassava flour and children’s CAC scores, assuming the relationship between cyanogen content and CAC scores is linear over the range of the cyanide content found in cassava flour [5]. It should also be noted that this unadjusted coefficient is not statistically significant after controlling the variables in the multivariable analysis. More studies are needed to understand the dose-response relationship between cyanide exposure and children’s intellectual performance.

Prevention measures can help eradicate the disease associated with cassava-dominant diet in konzo-affected areas. Programs on distributing less toxic varieties of cassava and disseminating new processing/detoxification of cassava roots before consumption have been introduced in some African countries to reduce the cyanide content to safe levels. The National Nutrition Program (PRONANUT) is playing a crucial role in coordinating nutrition governance in DRC. PRONANUT has taken the lead in sponsoring the wetting method for konzo prevention in Kahemba health zone. PRONANUT is dedicated to expanding their support for konzo prevention through programmatic and policy dissemination across the country. In some villages of Kahemba health zone, educational efforts of interventions are being implemented to reduce the presence of cyanogens in the diet of at-risk populations, including the wetting method intervention in conjunction with traditional methods to reduce cyanogens, which has been repeatedly demonstrated to reduce cyanogenic levels in poorly processed cassava flour to safe levels [29]. Additionally, certain traditional green leafy vegetables such as *Salacia pynaerti* and *Tetrochirdium congolenses* are associated with antioxidant activities, which are essential for the detoxification of cyanogen glycosides [39]. Taken together, these interventions can help reduce disease burden from a foodborne toxin relevant to Central Africa, which has shown long-term impacts to children’s cognitive health.

## Conclusion

The burden of cognitive impairment in Central African children who eat a monotonous cassava diet far exceeds that of konzo as a paralytic disease; therefore, substantially increases the estimate of burden of foodborne disease from cassava cyanide. Important to note is that children are a vulnerable population with typically little choice in diet, who may suffer lifelong consequences. In addition, prevention interventions and educational efforts are needed to reduce the presence of cyanogens in the diet of populations with konzo. With a more comprehensive estimate of disease burden from cassava cyanide exposure, we will have improved policy justification for the cost-effectiveness of interventions, including educational efforts and simple household-based methods, to make cassava consumption safer for at-risk children in DRC and other rural communities in Central Africa.
